# Characterization, Identification, and Antioxidant Mechanism of Antioxidant Peptides From *Schisandra chinensis* Based on Peptidome and Molecular Docking/Dynamics

**DOI:** 10.1002/fsn3.72028

**Published:** 2026-06-14

**Authors:** Shuo Zheng, Xingyu Xiao, Yi Li, Tong Su, Qinchuan Lv, Jiayuan Fang, Linlin Hao, Huayi Lu

**Affiliations:** ^1^ College of Animal Science Jilin University Changchun Jilin China; ^2^ Department of Ophthalmology, the First Affiliated Hospital of USTC, Division of Life Sciences and Medicine University of Science and Technology of China Hefei China

**Keywords:** antioxidant peptides, Keap1, molecular docking, molecular dynamics, *Schisandra chinensis*

## Abstract

*Schisandra chinensis*, a traditional Chinese medicine‐food homologous plant, is widely used for alleviating oxidative stress induced damage in hepatocytes and neurons due to its antioxidant activity. However, significant gaps remain in the research regarding the sequence, structure, and antioxidant mechanism of its bioactive peptides. Herein, we aimed to evaluate the antioxidant capacity of *S. chinensis* peptides (SCP), screen the optimal bioactive peptide sequences, and elucidate underlying antioxidant mechanisms. The results demonstrated that SCP exhibited potent free radical scavenging activity, with scavenging rates of over 90% and 50% against DPPH and ABTS radicals at 10 mg/mL, respectively. At 100 μg/mL, SCP restored the levels of ROS and MDA in UVB‐induced HaCaT to those of the undamaged control group. Meanwhile, SCP maintained over 65% DPPH radical scavenging activity under various physicochemical conditions and exhibited excellent biosafety with a hemolysis rate below 0.8% at a high concentration of 8 mg/mL. In total, 63 bioactive peptides were identified through peptidome and bioinformatics analyses, and 8 high affinity peptides for DPPH and ABTS were further screened out. FF4, FW4, and IL6 were capable of forming stable complexes with Keap1, primarily driven by hydrogen bonds and hydrophobic interactions, as revealed by molecular docking and molecular dynamics simulations. Specifically, the Keap1‐IL6 complex showed the most stable conformation, with DPPH and ABTS radical scavenging rates above 50% at 10 mg/mL and hemolytic activity below 10%. Collectively, these findings provide a scientific basis for clarifying the molecular mechanism of SCP in regulating antioxidant pathway and facilitating its subsequent industrialization.

## Graphical abstract

*Schisandra chinensis* peptides (SCP) exhibited potent in vitro antioxidant activity and repaired UVB‐induced oxidative damage in HaCaT cells, while demonstrating excellent stability and biosafety. SCP also activated the Keap1/Nrf2 pathway, with IFSPWL identified as the key bioactive peptide that forms the most stable Keap1 complex via integrated peptidomics and molecular docking/dynamics. These results highlight the potential of SCP, especially IFSPWL, as natural antioxidants for functional foods and skincare applications.

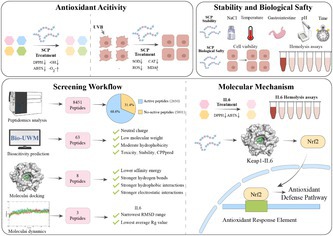

## 1 Introduction

The dynamic balance between oxidation and antioxidation constitutes a core mechanism for sustaining human health. When the organism is exposed to environmental stress or metabolic disorders, reactive oxygen species (ROS) accumulate excessively, which in turn triggers lipid peroxidation chain reactions to disrupt cell membrane fluidity, induces protein carbonylation modifications leading to the loss of enzyme activity, and even causes DNA strand breaks and base mutations (Schieber and Chandel 2014; Zhu et al. 2024). Cutaneous oxidative damage triggered by ultraviolet B (UVB) irradiation is a direct pathological consequence of sustained oxidative stress, culminating in skin erythema, photoaging, and even skin cancer (Fernandes et al. 2023; Wei et al. 2024). Exogenous supplementation of antioxidants is an important strategy to alleviate oxidative stress in the organism. Synthetic antioxidants such as butylated hydroxytoluene carry potential hepatotoxic and carcinogenic risks (Xu et al. 2021), while natural antioxidants like vitamins and plant polysaccharides, despite their high biocompatibility, suffer from inferior stability and low bioavailability (Al‐Madhagi and Masoud 2024; Zhu et al. 2022). In recent years, studies have confirmed that bioactive peptides, as a class of small molecule substances with both potent antioxidant activity and high biological safety, represent a highly promising alternative antioxidant for practical applications (Xu et al. 2024).

The antioxidant activity of peptides is dependent on the composition and sequence distribution of amino acids, with hydrophobic (Leu, Val), aromatic (Tyr, Trp), and sulfur‐containing (Cys) amino acids serving as the core functional residues (Chi et al. 2015). These residues enable antioxidant peptides to exert their effects through a dual mechanism that involves direct free radical scavenging and indirect antioxidant signaling pathway regulation. The peptides not only activate functional groups such as phenolic hydroxyls and sulfhydryls to effectively block oxidative chain reactions (Ndayiragije et al. 2023; Preczenhak et al. 2019), but also form intermolecular interactions including hydrogen bonds, hydrophobic effects, and π‐cation bonds with Keap1 protein to activate the intracellular expression of superoxide dismutase (SOD) and catalase (CAT) and other antioxidant enzymes (Yi et al. 2020).

*Schisandra chinensis*, abundant in lignins, flavonoids, volatile oils and other bioactive components, has been extensively investigated and applied in neuroprotection, anti‐inflammation, anti‐cancer and antioxidant fields (Yan et al. 2025; Zhang et al. 2018). Additionally, it contains a high content of amino acids with a large proportion of antioxidant active amino acids such as glutamic acid and cysteine (Egbujor et al. 2024; Yang and Yuan 2021), thus emerging as a promising resource for natural antioxidant peptides. Studies have confirmed that *S. chinensis* peptides (SCP) exhibit potent antioxidant activities. SCP effectively activates endogenous antioxidant enzymes such as CAT, SOD and GSH‐Px to protect hepatocytes and macrophages from H_2_O_2_‐induced oxidative damage (Ha‐Rim et al. 2021; Wang, Zhang, et al. 2025), and directly scavenges DPPH, hydroxyl radicals and ABTS free radicals (Liu et al. 2021; Zagórska‐Dziok et al. 2022). However, current research is still limited to the activity evaluation of crude SCP hydrolysates, with neither in‐depth analyses of the specific sequences, structural characteristics, and action mechanisms of the active peptides nor sufficient explorations in skin antioxidation application.

Peptidome, molecular dynamics (MD) simulation and other bioinformatic analysis have provided efficient strategies for antioxidant peptide research. A large number of studies have achieved high throughput screening of antioxidant peptides via peptide sequence identification and bioinformatic tools, involving the prediction of biological activities, cell‐penetrating ability, toxicity and other properties, which effectively improves the screening efficiency of antioxidant peptides (Liu et al. 2025; Xiao et al. 2025).

This study focuses on the exploration and mechanistic elucidation of SCP. (1) The SCP antioxidant capacity was evaluated by measuring antioxidant activity in vitro and repair efficacy against UVB‐induced oxidative damage in HaCaT cells. (2) Novel antioxidant peptides were identified and the sequence characteristics were analyzed using peptidome and bioinformatic approaches. (3) The molecular mechanisms underlying the Keap1‐mediated antioxidant effects of key peptide fragments (FW4, FF4, IL6) were verified through molecular docking and MD simulation techniques. This work is expected to provide a scientific basis for the application of SCP in functional foods and antioxidant preparations.

## 2 Materials and Methods

### 2.1 Materials and Reagents

SCP, a commercial plant‐derived peptide product in powder form, was extracted from the dried fruits of *S. chinensis* (Turcz.) Baill, which was purchased from Shandong Dashu Co. Ltd. (Shandong, China) in 2025. IFSPWL (IL6) were synthesized by SynPeptide Co. Ltd. (Nanjing, China). For all experiments, the peptide powder was dissolved in different solvents as required. All assay kits related to antioxidant indicators were purchased from Nanjing Jiancheng Bioengineering Institute (Nanjing, China), including 1,1‐diphenyl‐2‐picrylhydrazyl (DPPH), 2,2′‐azino‐bis 3‐ethylbenzothiazoline‐6‐sulphonic acid diammonium salt (ABTS), hydroxyl radical, superoxide anion, superoxide dismutase (SOD), malondialdehyde (MDA), catalase (CAT) and ROS. Glutathione (GSH) was purchased from Solarbio Science & Technology Co. Ltd. (Beijing, China). Pepsin (3000 U/mg) and trypsin (250 U/mg) were purchased from Yuanye Biotechnology Co. Ltd. (Shanghai China). The Cell Counting Kit‐8 (CCK‐8) assay kit was purchased from Invigentech Corporation (CA, USA). HaCaT cells, a human epidermal keratinocyte cell line, were preserved in our laboratory. All other chemicals and reagents not mentioned herein were analytical grade and commercially available. All commercial assay kits were used according to the manufacturers' protocols.

### 2.2 *Schisandra chinensis* Peptide Antioxidant Activity

The free radical scavenging rates were determined at a range of SCP concentrations or IL6. The half‐maximal inhibitory concentration (IC_50_) was calculated to characterize the antioxidant activity of SCP. Glutathione (GSH) at the same concentration as SCP was used as the positive control.

### 2.3 Antioxidant Capacity of SCP on UVB‐Induced Damaged HaCaT Cells

#### 2.3.1 Cell Viability

HaCaT cells were exposed to UVB irradiation at 40 mJ/cm^2^, and then cultured in DMEM supplemented with SCP for 24 h. After incubation, cell viability was determined by CCK‐8 assay.

#### 2.3.2 SOD, CAT, and MDA Levels

HaCaT cells were treated as described in Section 2.3.1 and then measured Intracellular SOD, CAT activity, and MDA content.

#### 2.3.3 ROS Fluorescence

HaCaT cells were treated as described in Section 2.3.2. Subsequently, cells were incubated with 10 μM DCFH‐DA at 37°C for 0.5 h, then washed three times with PBS. Intracellular fluorescence signals were visualized under a fluorescence microscope, and the relative fluorescence intensity was analyzed using ImageJ.

### 2.4 SCP Stability

#### 2.4.1 pH, Heat, and Salt Treatments

The stability of SCP antioxidant activity under pH, heat, and salt stress conditions was evaluated according to the method established by Priyanka Singh Rao et al. (2020). Briefly, for pH level evaluation, the pH levels of five aliquots from the 10 mg/mL SCP solution were adjusted to 3, 5, 7, 9, and 11 using 50 mM HCl and 50 mM NaOH solutions, respectively. After oscillating at room temperature (25°C) for 2 h, the pH of each group was readjusted to 7, and then the DPPH radical scavenging rate was measured. For heat treatment evaluation, 10 mg/mL SCP solutions were incubated at 25°C, 40°C, 60°C, 80°C, and 100°C for 2 h, and then rapidly cooled to room temperature on ice to measure the DPPH radical scavenging rate. For salt treatment, 10 mg/mL SCP solutions were prepared using 0, 2, 4, 6, and 8 mg/mL NaCl solutions as the solvent. After incubating at room temperature for 1 h, the DPPH radical scavenging rate was measured.

#### 2.4.2 Gastrointestinal Digestion

Simulated pancreatic juice (SPJ) and simulated gastric juice (SGJ) were prepared according to the method established by Lo Curto et al. (2011) with minor modifications. Briefly, SPJ was prepared at pH 6.8 with 2500 U/mL, consisting of 50 mM KH_2_PO_4_, 50 mM NaOH, and 250 U/mg trypsin. SGJ was prepared at pH 1.2 with 9600 U/mL, consisting of 125 mM NaCl, 50 mM HCl and 3000 U/mg pepsin. The 10 mg/mL SCP solutions were prepared using SPJ and SGJ, respectively, as the solvent. After incubating at 37°C with agitation at 100 rpm for 2 h, the SCP solutions were neutralized to pH 7.0 and heated in a 95°C water bath for 10 min to inactivate the enzymes. Finally, the gastrointestinal stability of SCP was evaluated by measuring the DPPH radical scavenging activity of the undigested control, the SPJ‐digested group, and the SGJ‐digested group.

#### 2.4.3 Time

Five aliquots from 10 mg/mL SCP solution were prepared and stored at room temperature under a 12 h/12 h light–dark cycle for 0, 1, 3, 5, and 7 days, respectively. DPPH radical scavenging rate was measured to evaluate the temporal stability of SCP.

### 2.5 SCP Biosafety

#### 2.5.1 Cytotoxicity

HaCaT cells were cultured in DMEM medium containing 50, 75, 100, 125, 250, and 500 μg/mL SCP for 24 h. Cell viability was measured using the CCK‐8 assay to evaluate the cytotoxic effect of SCP.

#### 2.5.2 Hemolytic Assay

Fresh mouse red blood cells (RBCs) were centrifuged at 3000 rpm for 10 min at 4°C. The pellet was washed three times with PBS and resuspended to prepare a 1% (v/v) RBC. Equal volumes of 1, 2, 4, and 8 mg/mL SCP were mixed with the RBC, followed by incubation at 37°C, 200 rpm for 1 h. After incubation, the absorbance was measured at 570 nm. PBS served as the negative control, and 0.1% Triton X‐100 as the positive control. The hemolytic activity of SCP or IL6 was calculated using the formula (Li et al. 2017): Hemolysis rate (%) = (OD_(peptide)_ − OD_(PBS)_)/(OD_(0.1% Triton X‐100)_ − OD_(PBS)_) × 100%.

### 2.6 SCP Molecular Weight

The molecular weight distribution of SCP was determined using high‐performance liquid chromatography (HPLC) (Waters, USA). The mobile phase consisted of acetonitrile/water/trifluoroacetic acid (40:60:0.01, v/v/v) at a flow rate of 0.5 mL/min, with detection at 220 nm. Different molecular weights were used as gel filtration chromatography standards. The molecular weight distribution was established based on the correlation between the retention time (Rt) and the logarithm of relative molecular weight (lg(M)).

### 2.7 Identification and Screening of SCP Sequences

#### 2.7.1 Peptidome

The peptide composition of SCP was performed by liquid chromatography–tandem mass spectrometry (LC–MS/MS) on a VANQUISH NEO Nano LC system coupled with a Q Exactive HF‐X mass spectrometer (Thermo Fisher Scientific, USA). The column was equilibrated prior to analysis, with a column temperature of 55°C and a flow rate of 0.3 μL/min. Mobile phase A consisted of ultrapure water with 0.1% (v/v) formic acid, and mobile phase B was 80% (v/v) acetonitrile containing 0.1% (v/v) formic acid. Mass spectra were acquired in positive electrospray ionization mode with a spray voltage of 2.2 kV using a data‐dependent acquisition strategy, in which the top 20 precursor ions were subjected to collision‐induced dissociation per full scan. After data acquisition, raw data were processed with Proteome Discoverer 3.0.

#### 2.7.2 Database‐Based Screening of Antioxidant Peptide Sequences

PeptideRanker (http://distilldeep.ucd.ie/PeptideRanker/) was used to predict the bioactivity of peptide sequences with a screening threshold set at 0.9. BIOPEP‐UWM (https://biochemia.uwm.edu.pl/biopep/start_biopep.php) was used to predict the biological activities of candidate bioactive peptides and screen out the peptide fragments with antioxidant activity.

#### 2.7.3 Physicochemical Property Prediction of Antioxidant Peptide Sequences

The molecular formula and primary structure of the peptides were predicted using the online platform (https://www.allpeptide.com/). Peptide length, net charge, relative molecular weight, isoelectric point, and hydrophobicity were analyzed via PepDraw (https://pepdraw.com/). The toxicity was evaluated using the ToxinPred database. Additionally, the antioxidant potential of the identified peptides was further verified through AnOxPP‐1.0 (http://www.cqudfbp.net/AI‐Tools/AnOxPP/), and the BPP‐Tool (http://www.cqudfbp.net/bitterPrediction/tools/DataInput.jsp) was employed to assess the bitterness. The water solubility of peptides was determined using PepCalc.com (https://www.pepcalc.com/). Furthermore, the cleavage sites of the peptides for pepsin, trypsin, and chymotrypsin were predicted using the online platform (https://www.novopro.cn/tools/protease‐digestion‐tool.html).

### 2.8 Molecular Docking

PubChem (https://pubchem.ncbi.nlm.nih.gov/) provided the structural data of DPPH (CID:2735032) and ABTS (CID:5360881), and RCSB PDB (https://www.rcsb.org/) provided the structure data of human Keap1 (PDB:2FLU). The target peptide was modeled on the online platform (https://cloud.yinfotek.com). Keap1 crystal structure was optimized via AutoDockTools 1.5.6 through removing water molecules and native ligands, adding hydrogen atoms, assigning charges and defining atomic types. Two molecular docking assays were conducted: peptide as receptor with DPPH/ABTS as ligands, and peptide as ligand with Keap1 (docking site: *x* = 5, *y* = 9, *z* = 2) as receptor, with the binding energy of peptide‐ligand complexes calculated in each assay. Docking results were finally visualized and analyzed by PyMOL 2.2.0 and Discovery Studio 2018.

### 2.9 Molecular Dynamics (MD) Simulation

The conformational structure of the peptide‐Keap1 complex was used as the initial model for MD simulations via GROMACS 2021.4 software. The ff19SB force field was applied for parameter assignment, and the TIP3P model was used to set up a simulation box a side length 1.0 nm larger than the complex. Na^+^ and Cl^−^ ions were introduced to achieve overall charge neutrality. After energy minimization, 100 ps NVT and 100 ps NPT equilibration were performed at 300 K and 1 bar. A 100 ns production MD simulation was carried out, with atomic coordinates saved every 2 ps and trajectory files recorded every 10 ps. GROMACS tools were used for simulation analysis, calculating the root‐mean‐square deviation (RMSD), root‐mean‐square fluctuation (RMSF), number of hydrogen bonds and solvent‐accessible surface area (SASA) to evaluate its conformational stability and interaction mode.

### 2.10 Statistical Analysis

Data are presented as mean ± standard deviation (SD, *n* = 3). Differences among group means were evaluated using one‐way analysis of variance (ANOVA). *p*‐value < 0.05 was considered statistically significant, denoted as *p < 0.05. Different letters indicate significant differences among groups (*p* < 0.05). All data analyses and visualizations were performed using GraphPad Prism 9.0.

## 3 Results

### 3.1 Radical Scavenging Activities of SCP

**FIGURE 1:**
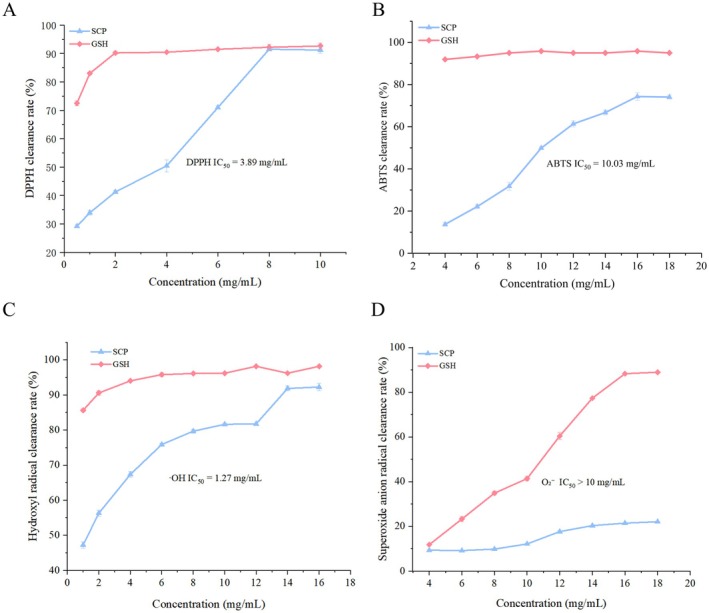
SCP in vitro antioxidant activity. (A–D) The scavenging rates of SCP on DPPH radicals (A), ABTS radicals (B), hydroxyl radicals (C), and superoxide anions (D).

To evaluate the antioxidant activity of SCP, four distinct free radical scavenging activities were determined, with GSH serving as the positive control. SCP exhibited dose‐dependent scavenging activities against DPPH, ABTS radicals, and hydroxyl radicals, with relatively weak activity against superoxide anion radicals, as shown in Figure 1. Notably, at 8 mg/mL, the DPPH radical scavenging capacity of SCP was equivalent to that of GSH (*p* > 0.05). Moreover, SCP had IC_50_ values of only 3.89 mg/mL and 1.27 mg/mL for DPPH free radicals and hydroxyl radicals, respectively. Overall, SCP exhibited strong antioxidant activity.

### 3.2 Repair Effects of SCP Against UVB‐Induced Oxidative Damage in HaCaT Cells

**FIGURE 2:**
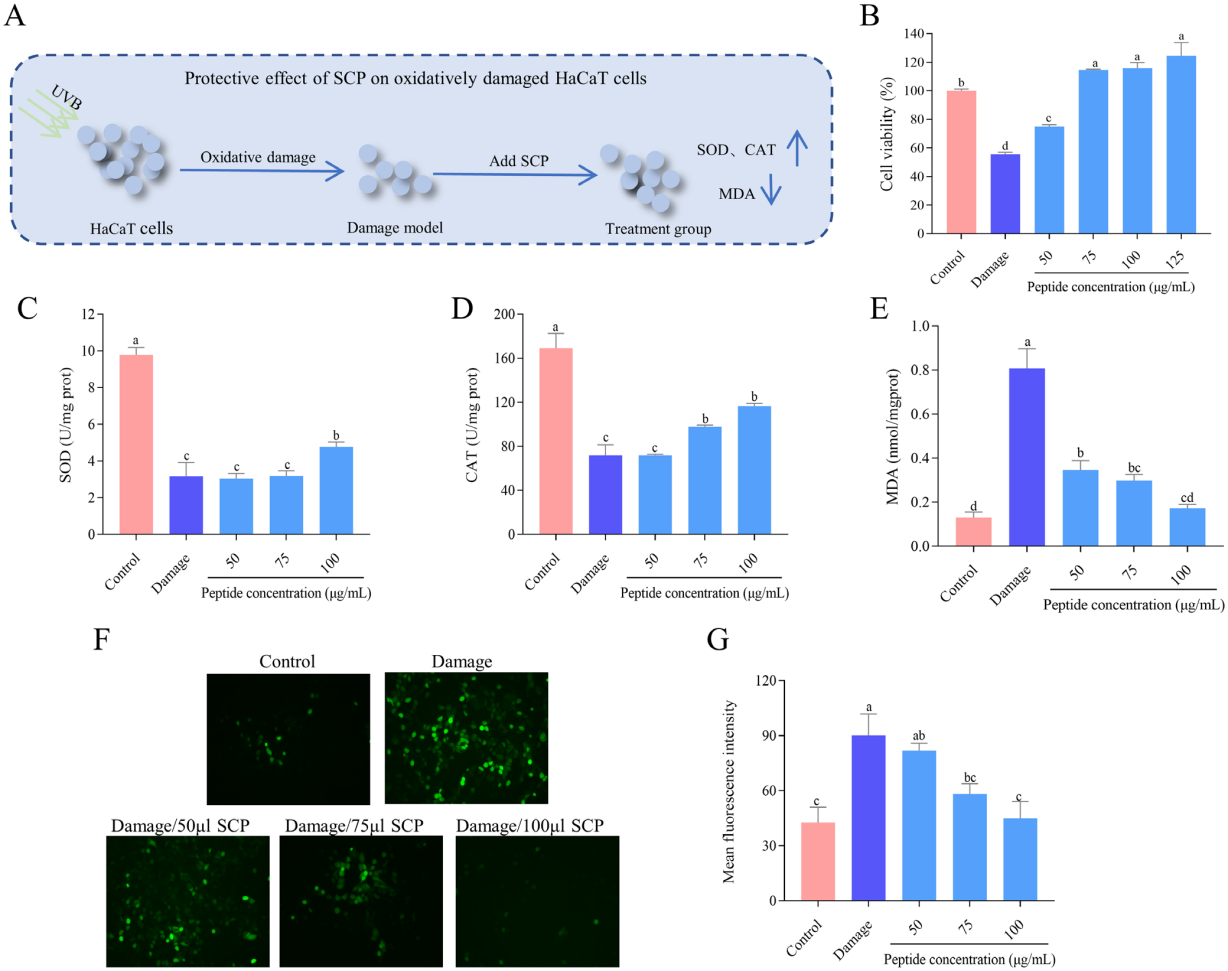
SCP repair capacity for UVB‐induced oxidatively damaged HaCaT cells. (A) Schematic of SCP repairing oxidatively damaged HaCaT cells. (B–E) Effects of SCP at different concentrations on cell viability (B), SOD activity (C), CAT activity (D), and MDA content (E) in UVB‐induced HaCaT cells. (F–G) ROS content (F) and fluorescence intensity analysis (G) of HaCaT cells under different treatments.

UVB irradiation triggers excessive ROS generation, impairs the skin defense system, reduces SOD and CAT activities, and triggers lipid peroxidation, resulting in elevated MDA levels (Liu et al. 2024). Accordingly, we investigated the protective effect of SCP against UVB‐induced oxidative damage in HaCaT cells (Figure 2A). Results showed that cell viability in the damage group was significantly lower than that in the control group (*p* < 0.05), confirming the successful establishment of the model. SCP elevated the average cell viability from 55.59% to 114.67% and 124.46% at concentrations of 75 μg/mL and 125 μg/mL, respectively, which was even higher than that of the control group (Figure 2B). Meanwhile, SOD and CAT activities recovered in a dose‐dependent manner after SCP treatment, reaching 48.70% and 68.96% of the control group at 100 μg/mL (Figure 2C,D). Consistently, SCP also dose‐dependently reduced the content of MDA, the marker of lipid peroxidation, and no significant difference was observed between the 100 μg/mL SCP group and the control group (*p* > 0.05) (Figure 2E). As shown in Figure 2F, the average fluorescence intensity in the damage group was much higher than that in the control group (*p* < 0.01), indicating massive ROS accumulation. Compared with the damage group, intracellular ROS levels decreased in a dose‐dependent manner after SCP treatment, with ROS levels quenched by 8.24%, 31.91%, and 45.22%, respectively (Figure 2G). These results indicate that SCP exerts a protective effect against UVB‐induced oxidative damage in HaCaT cells via enhancing antioxidant enzyme activities and scavenging intracellular ROS.

### 3.3 Stability and Biological Safety of SCP

**FIGURE 3:**
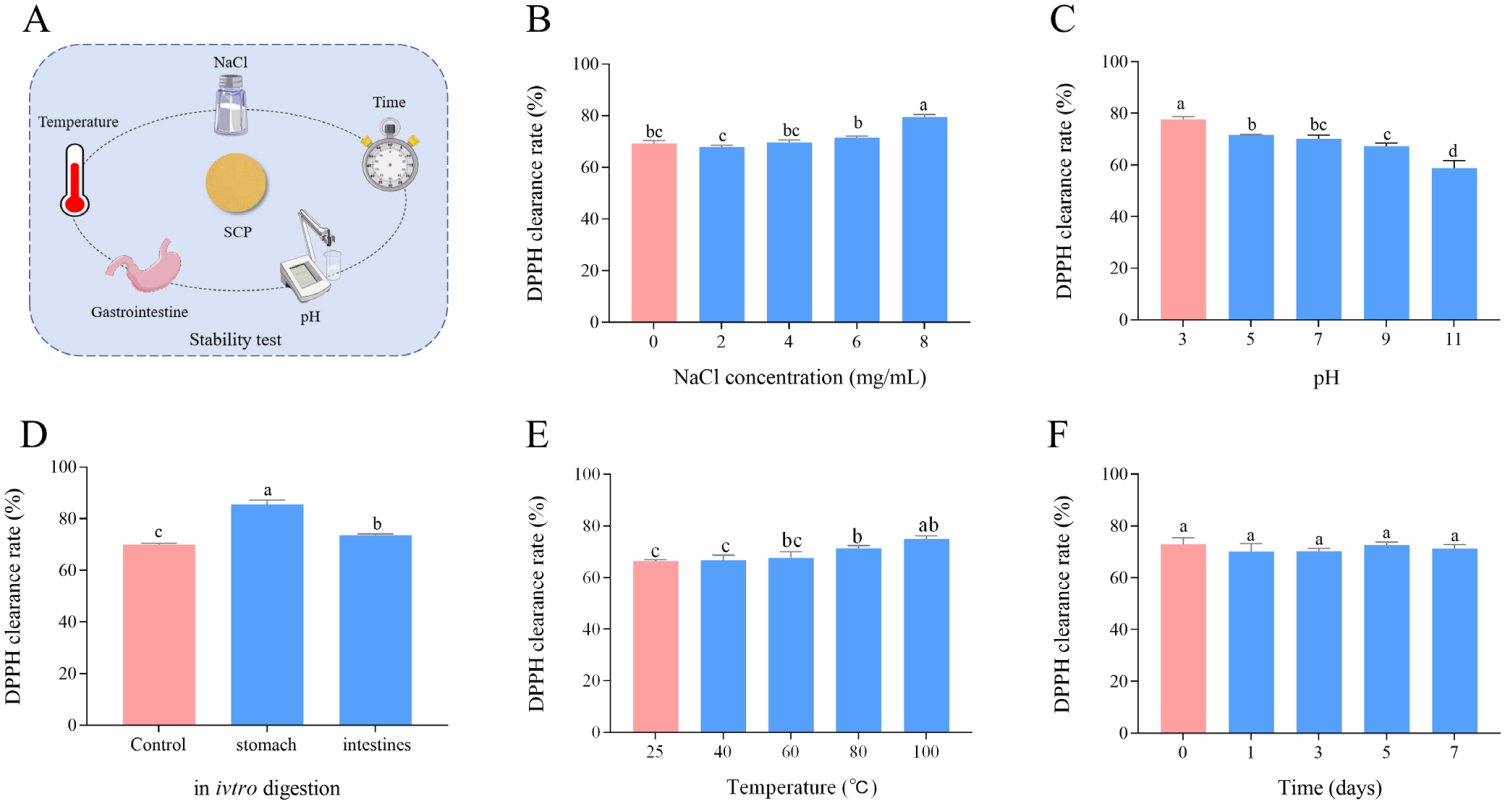
SCP stability. (A) Schematic of SCP stability determination. (B–F) DPPH radical scavenging activity of SCP under different NaCl concentrations (B), temperatures (C), digestive enzymes (D), pH values (E), and storage times (F).

Bioactive peptides tend to lose their activity easily due to complex conditions in processing environments, especially pH, salt ions, and temperature, and human gastrointestinal digestion before being processed into functional foods or absorbed (Pei et al. 2022). Therefore, we evaluated the stability of SCP under different conditions using DPPH scavenging rate as the indicator (Figure 3A). The DPPH scavenging rate of SCP rose to over 73.63% with increasing NaCl concentration and temperature (Figure 3B,C). Meanwhile, the DPPH scavenging rate of SCP was enhanced by 15.01% in simulated gastric juice and by 6.46% at pH 3 relative to that measured under neutral pH conditions, in contrast to many other peptides that often lose activity during gastrointestinal digestion or in acidic environments (Figure 3D,E). Moreover, it showed no significant difference from the initial value after 7 days of storage (*p* > 0.05) (Figure 3F). Overall, SCP exhibited good stability.

**FIGURE 4:**
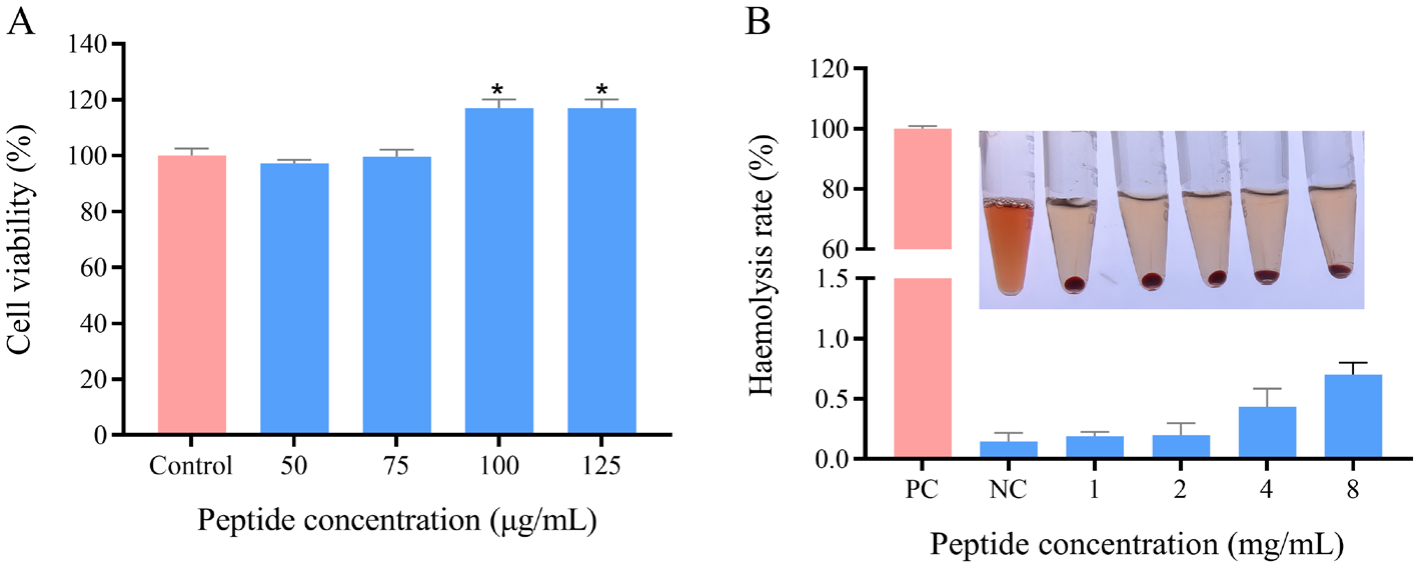
SCP biological safety. (A) Cytotoxicity of SCP on HaCaT cells. (B) Hemolytic activity of SCP on mouse red blood cells.

Given the importance of biosafety for bioactive peptides in practical applications, we further evaluated the biosafety of SCP via cell viability and hemolysis assays. As the concentration of SCP increased from 50 μg/mL to 125 μg/mL, cell viability significantly enhanced (*p* < 0.05), indicating that SCP not only had no cytotoxicity but also exerted a cell proliferation‐promoting effect (Figure 4A). In the hemolysis assay, 0.1% Triton X‐100 (PC) was used as the positive control and normal saline (NC) as the negative control. As shown in Figure 4B, the hemolysis rate of SCP was much lower than that of PC, and its hemolytic activity remained below 0.8% at concentrations ranging from 1 to 8 mg/mL, confirming that SCP possessed good biosafety.

### 3.4 Molecular Weight Distribution of SCP

**FIGURE 5:**
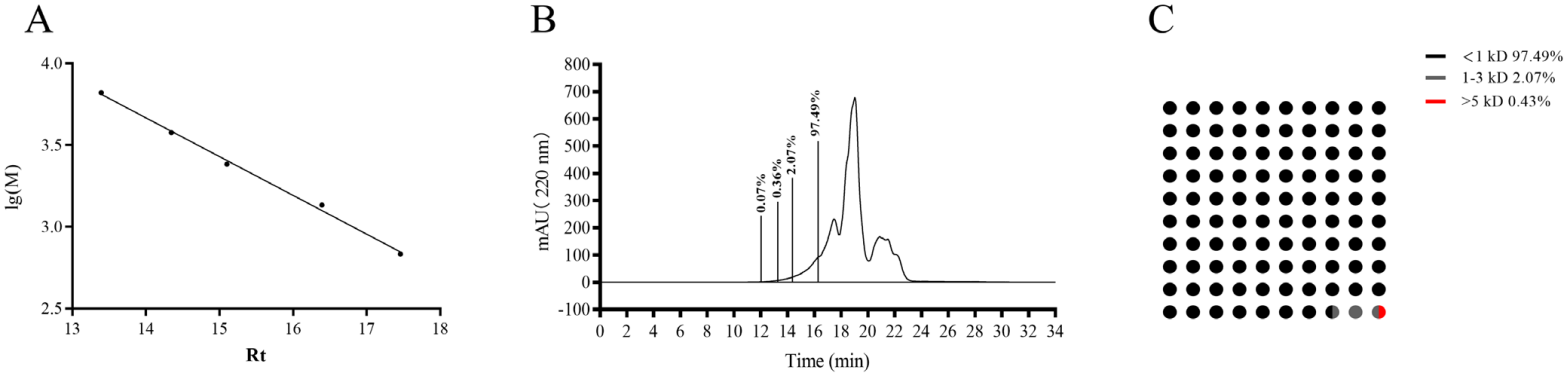
SCP molecular weight distribution. (A) Standard curve between elution time and molecular weight. (B) Chromatogram of SCP. (C) Molecular weight distribution of SCP.

Multiple studies have shown that peptides with MW < 1 kDa exhibit higher antioxidant activity (Zhou et al. 2024). To determine the MW distribution of SCP, a standard curve between elution time (Rt) and logarithm of molecular weight (lg(M)) was first established, confirming a linear relationship between the two parameters (Figure 5A). Further chromatogram analysis revealed that SCP had multiple elution peaks, indicating it consists of fractions with different MWs (Figure 5B). Specifically, SCP contained 97.49% peptides with MW < 1 kDa, while peptides with MW > 5 kDa only accounted for 0.43% (Figure 5C), demonstrating that SCP is dominated by low‐MW peptides.

### 3.5 Identification and Physicochemical Property Analysis of SCP Peptide Sequences

**FIGURE 6:**
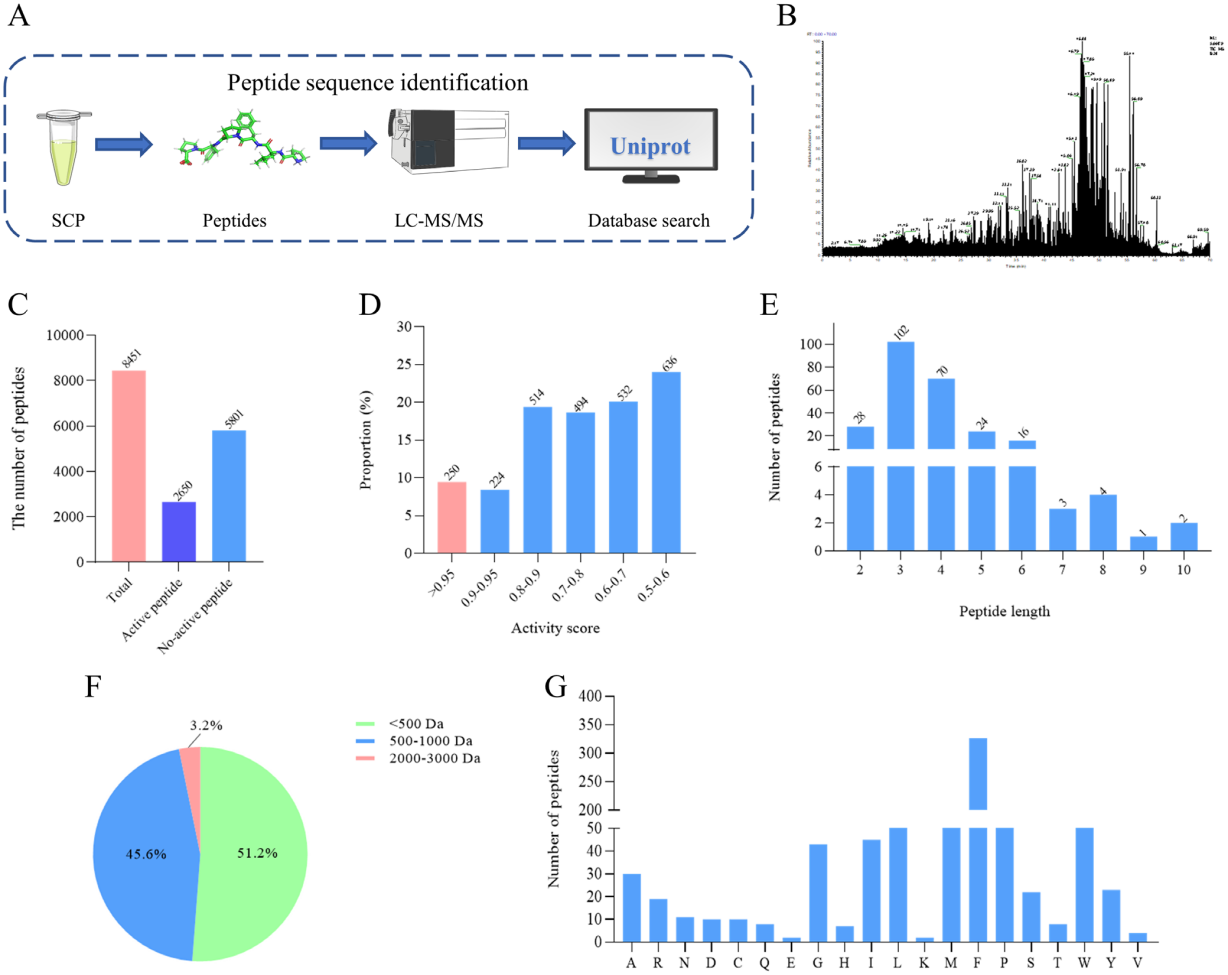
SCP peptide sequence identification and screening. (A) Schematic of peptidomics analysis. (B) LC–MS/MS total ion current chromatogram of SCP. (C) Statistics of bioactive peptides in SCP. (D–G) Peptide Ranker activity score distribution ratio (D), length distribution (E), molecular weight distribution (F), hydrophobic amino acid number distribution (G).

The peptide sequence identification and analysis of SCP were conducted as shown in Figure 6A. A total of 8451 peptides were identified in SCP via LC–MS/MS, including 2650 potential active peptides and 5801 non‐active peptides (Figure 6B,C). Among these, 250 peptides had a peptide ranker score higher than 0.95, accounting for 2.96% of the total peptides (Figure 6D). Further analysis of high‐score peptides showed that most peptide segments contained 2–6 amino acid residues, and peptide segments with more than 7 amino acid residues accounted for only 4% (Figure 6E). Moreover, 96.8% of these peptides had a molecular weight less than 1000 Da (Figure 6F), exhibiting the characteristics of high antioxidant activity peptides. In addition, the total number of hydrophobic amino acids contained in 250 high‐score peptide segments exceeded 600, including F (Phenylalanine), L (Leucine), I (Isoleucine), and A (Alanine) (Figure 6G).

In total, 63 peptides were further confirmed to have biological activity using Bio‐UWM (Table S1), and optimal peptides were predicted by physicochemical properties (Table S2). Most peptides carried a neutral charge, with pI ranging from 5.33 to 5.98, which helps SCP maintain structural stability in the biological environment, avoid unnecessary molecular aggregation, and interact with free radicals more smoothly. Meanwhile, these peptides also had low MW (< 1000 Da) and were classified as AnOxP, with high bioavailability and antioxidant stress capacity. All peptides except AFFCILLPF, AGGF, and AGFC had moderate hydrophobicity (2–8), balancing solubility and hydrophobic free radical binding efficiency, driven by residues like F (Phenylalanine) and L (Leucine). Although most peptides were bitter, it did not directly hinder antioxidant activity, and the distribution of pepsin/trypsin/chymotrypsin digestion sites indicated that these peptides could resist excessive hydrolysis during gastrointestinal transit, ensuring complete delivery to target sites. In addition, 21 peptides had good cell penetration ability (CPPpred > 0.1), and 4 peptides were soluble in water.

### 3.6 Screening and Potential Mechanism of SCP Based on Molecular Docking and Molecular Dynamics (MD) Simulation

#### 3.6.1 Molecular Docking of Antioxidant Peptides With DPPH and ABTS

To efficiently identify the most potent antioxidant peptide from 63 biological activity peptides, molecular docking was performed using stable DPPH and ABTS free radicals (Table S3). All peptides exhibited full binding ability to DPPH and ABTS free radicals; among them, FYPF and FYLPF showed relatively low docking binding energy values in both free radical systems, which were −4.5 kcal/mol and −4.3 kcal/mol, and −4.6 kcal/mol and −4.2 kcal/mol, for DPPH and ABTS respectively. In addition, intermolecular interactions such as hydrogen bonding and hydrophobic interactions also played a key role. Specifically, amino acid residues Trp4 and Phe6 in GISWGFL formed 2 hydrogen bonds with DPPH, while residues Ser3 and Trp5 in IFSPWL bound to ABTS through hydrophobic interactions. Furthermore, Phe, Trp, and Leu had higher occurrence frequency than that of other amino acid residues during the interaction of all peptides with the two free radicals, suggesting that these residues could serve as key active sites for SCP to scavenge free radicals. Based on the characteristics of antioxidant peptides and free radical scavenging capacity, FYPF, FLPW, FYLPF, ANWLPF, IFSPWL, FPFTYAMMLM, GPSFPIFI, and GISWGFL were selected as optimal antioxidant peptides.

#### 3.6.2 Functional Analysis of Amino Acid Residues in Antioxidant Peptides

**FIGURE 7:**
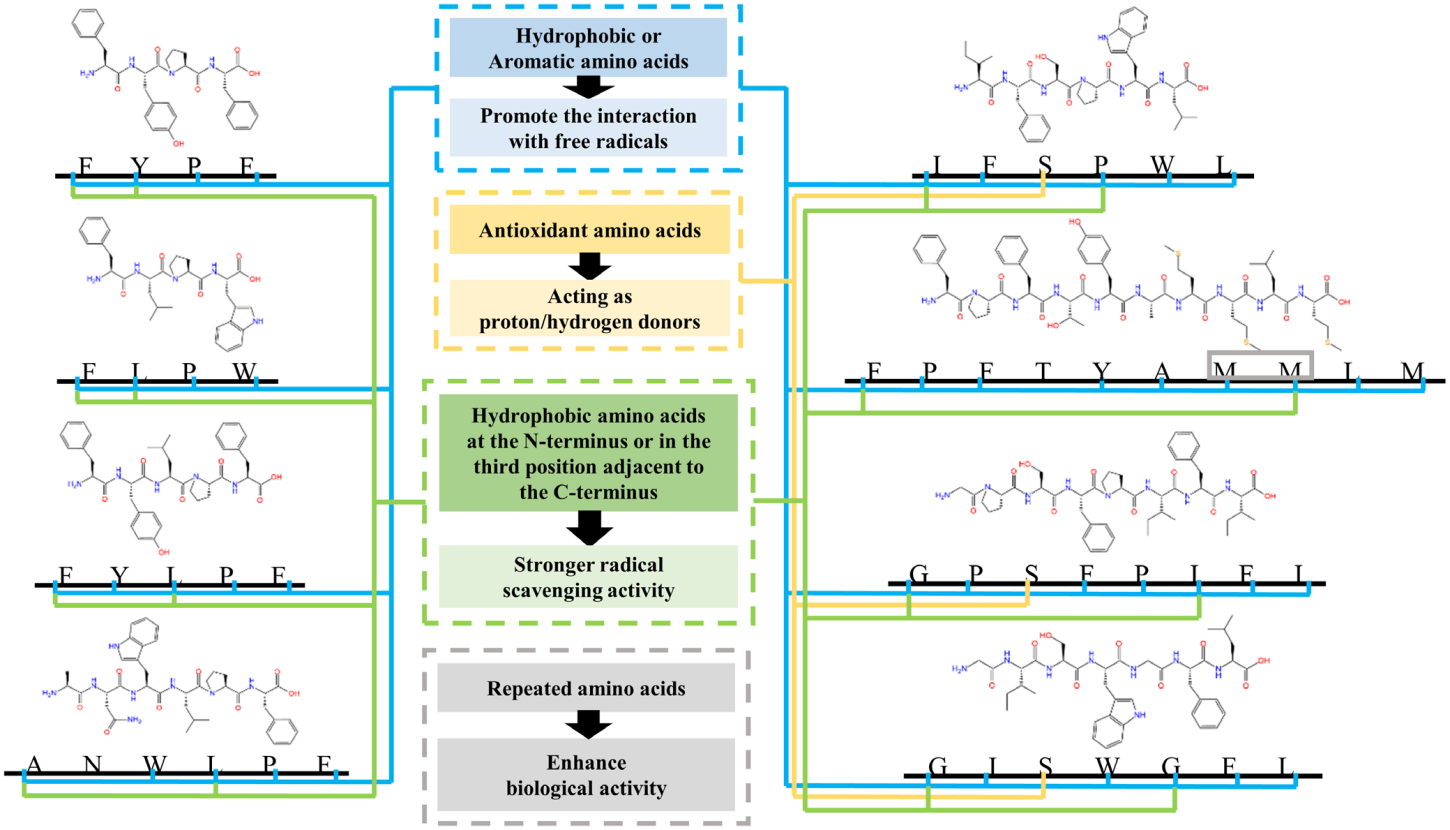
Antioxidant peptide structure–activity relationships of SCP. Different colors indicate distinct functional roles of amino acids.

To identify the structural features associated with antioxidant activity in the eight optimal peptides, the functional characterization of their amino acid residues was deeply analyzed (Figure 7). Hydrophobic or aromatic amino acids, such as F (Phenylalanine), I (Isoleucine), L (Leucine), and W (Tryptophan), in these peptides promote interaction with free radicals, thereby achieving effective scavenging. The antioxidant amino acids containing S (Serinein) in IFSPWL, GPSFPIFI, and GISWGFL act as proton/hydrogen donors, helping to neutralize free radicals. In addition, 8 peptides with hydrophobic amino acids located at the third position adjacent to the N‐terminal or C‐terminal enhance free radical scavenging activity. Moreover, repeated amino acids in FYPF, FYLPF, FPFTYAMMLM, GPSFPIFI, and GISWGFL further improve biological activity, reaffirming that FYPF, FLPW, FYLPF, ANWLPF, IFSPWL, FPFTYAMMLM, GPSFPIFI, and GISWGFL possess strong antioxidant potential.

#### 3.6.3 Molecular Docking of Antioxidant Peptides With Keap1

**FIGURE 8:**
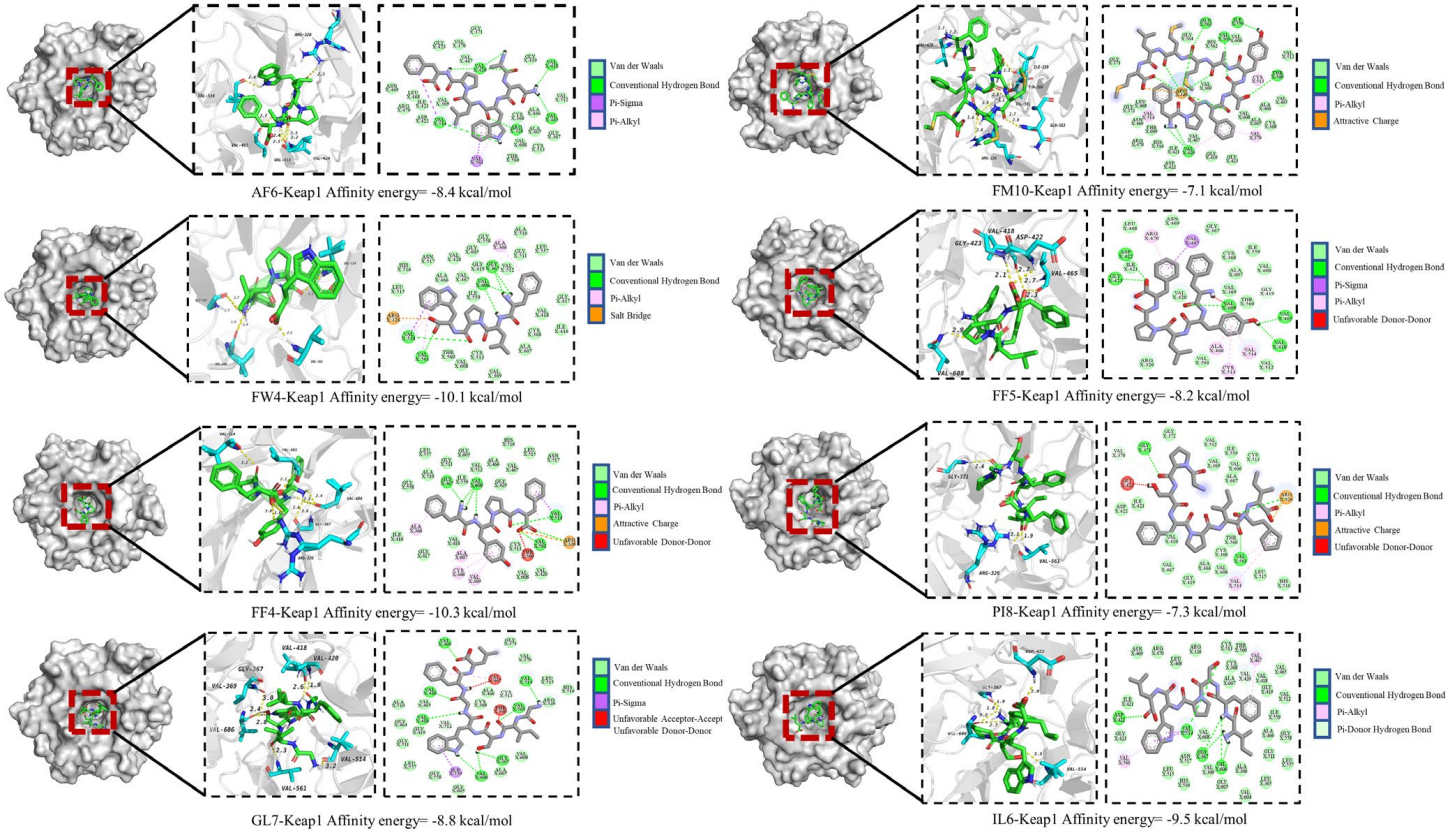
Molecular docking analysis between eight antioxidant peptides and Keap1.

The Keap1‐Nrf2 pathway serves as the core of the intracellular endogenous antioxidant defense system (Ma et al. 2023). Antioxidant peptides induce conformational changes in Keap1, thereby facilitating Nrf2 release to scavenge ROS. To investigate the potential mechanism of the 8 optimal antioxidant peptides, we further analyzed their binding energy and binding sites in the interaction with Keap1. The results demonstrated that all 8 peptides stably bound to the Keap1 domain, with binding energies ranging from −7.1 to −10.3 kcal/mol, indicating strong binding energy. Among these peptides, FF4 exhibited the strongest binding energy (−10.3 kcal/mol), followed by FW4 (−10.1 kcal/mol) and IL6 (−9.5 kcal/mol), suggesting that these three peptides possessed the optimal binding stability with Keap1 (Figure 8).

In terms of intermolecular forces, all 8 peptides formed extensive hydrophobic interactions within the Keap1 via van der Waals forces. A part of peptides could further form stronger hydrogen bonds, hydrophobic interactions (Pi‐Alkyl/Pi‐Sigma), and electrostatic interactions (Salt Bridge/Attractive Charge) with target residues, which synergistically enhanced their binding energy to the receptor. Among these interactions, hydrogen bonds represented the primary contributor to binding energy, accounting for 50%–87.5% of the total interactions excluding van der Waals forces, followed by hydrophobic interactions and electrostatic interactions (Figure 8). Specifically, in the van der Waals forces‐mediated interactions between the 8 peptides and Keap1, residues valine (VAL) and glycine (GLY) were the major contributors, with VAL‐418, VAL‐447 and GLY‐419 identified as the key residues with the highest frequency of occurrence. Notably, IL6 formed the largest number of van der Waals forces interactions. Hydrogen bond formation was also dependent on residues VAL, which accounted for 61.54% of the total hydrogen bonds formed by the 8 peptides; among these, VAL‐514 and VAL‐561 exhibited the highest frequency. Furthermore, FW4, FM10, FF4 and PI8 each formed 1 salt bridge with the residue ARG‐326 of the Keap1. FW4, FM10, FF4 and IL6 formed a relatively large number of interactions with Keap1. Importantly, FW4 and FF4 were capable of forming a considerable number of interactions with a minimal number of atoms (Table 1). Collectively, these findings demonstrate that the screened potential antioxidant peptides induce conformational changes in Keap1 through van der Waals forces, hydrogen bonds, hydrophobic interactions and electrostatic interactions, thereby disrupting the Keap1‐Nrf2 interaction and exerting antioxidant effects.

**TABLE 1:** Affinity energy and intermolecular forces between antioxidant peptides and Keap1.

Peptide	Abbreviation	Affinity energy (kcal/mol)	Number of Van der Waals	Number of hydrogen bonds	Number of hydrophobic interactions	Number of electrostatic interactions
ANWLPF	AF6	−8.7	19	5	1	—
FLPW	FW4	−10.1	23	4	1	1
FPFTYAMMLM	FM10	−7.6	22	6	3	1
FYLPF	FF5	−8.2	15	5	5	—
FYPF	FF4	−10.3	19	5	4	1
GISWGFL	GL7	−8.8	19	7	1	—
GPSFPIFI	PI8	−7.3	18	3	1	1
IFSPWL	IL6	−9.5	28	4	2	—

#### 3.6.4 Molecular Dynamics (MD) Simulation of Antioxidant Peptides With Keap1

**FIGURE 9:**
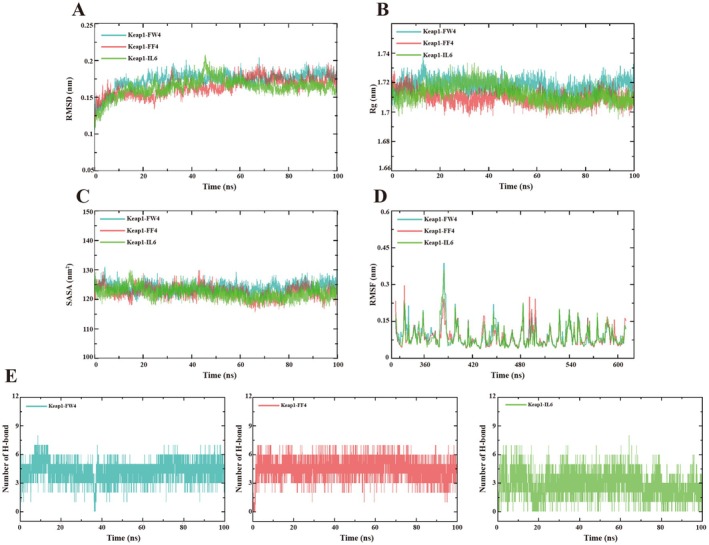
Molecular dynamics simulation analysis between three antioxidant peptides and Keap1. (A–C) RMSD (A), Rg (B) and SASA (C) variations of three complexes over simulation time. (D) RMSF profiles of Keap1 in complexes with FVA, F4, and IL6. (E) Number of hydrogen bonds between Keap1 and three peptides over simulation time.

Based on affinity energy and the number of interaction bonds, FW4, FF4, and IL6 were identified as the optimal potential antioxidant peptides. MD simulations were further performed to elucidate their binding characteristics and mechanisms with Keap1 at the atomic level. The results showed that the root‐mean‐square deviation (RMSD) of peptide‐Keap1 complexes in each system stabilized after 10 ns, fluctuating within the range of 0.15–0.2 nm, indicating high complex stability (Figure 9A). The radius of gyration (Rg), which characterizes the compactness of the complex structure (Choudhury and Dasmahapatra 2025), fluctuated slightly between 1.7 and 1.74 nm in all systems, suggesting strong affinity between the peptides and Keap1 (Figure 9B). Meanwhile, the solvent accessible surface area (SASA) of complexes in each system decreased gradually, which reflects reduced exposure to water molecules and a tendency toward a compact, stable conformation. Among them, the Keap1‐IL6 complex exhibited the most significant SASA reduction, followed by Keap1‐FF4 and Keap1‐FW4 (Figure 9C). The root‐mean‐square fluctuation (RMSF) reflects amino acid residue flexibility (Guo et al. 2026), and the RMSF values of all complexes were below 0.45 nm, with only slight fluctuations observed at residues 360–390 in FW4 and IL6, confirming high stability of the three complexes (Figure 9D). In addition, the number of hydrogen bonds formed between the three peptides and Keap1 is shown in Figure 9E, indicating stable hydrogen bonding interactions that are consistent with molecular docking analysis.

**FIGURE 10:**
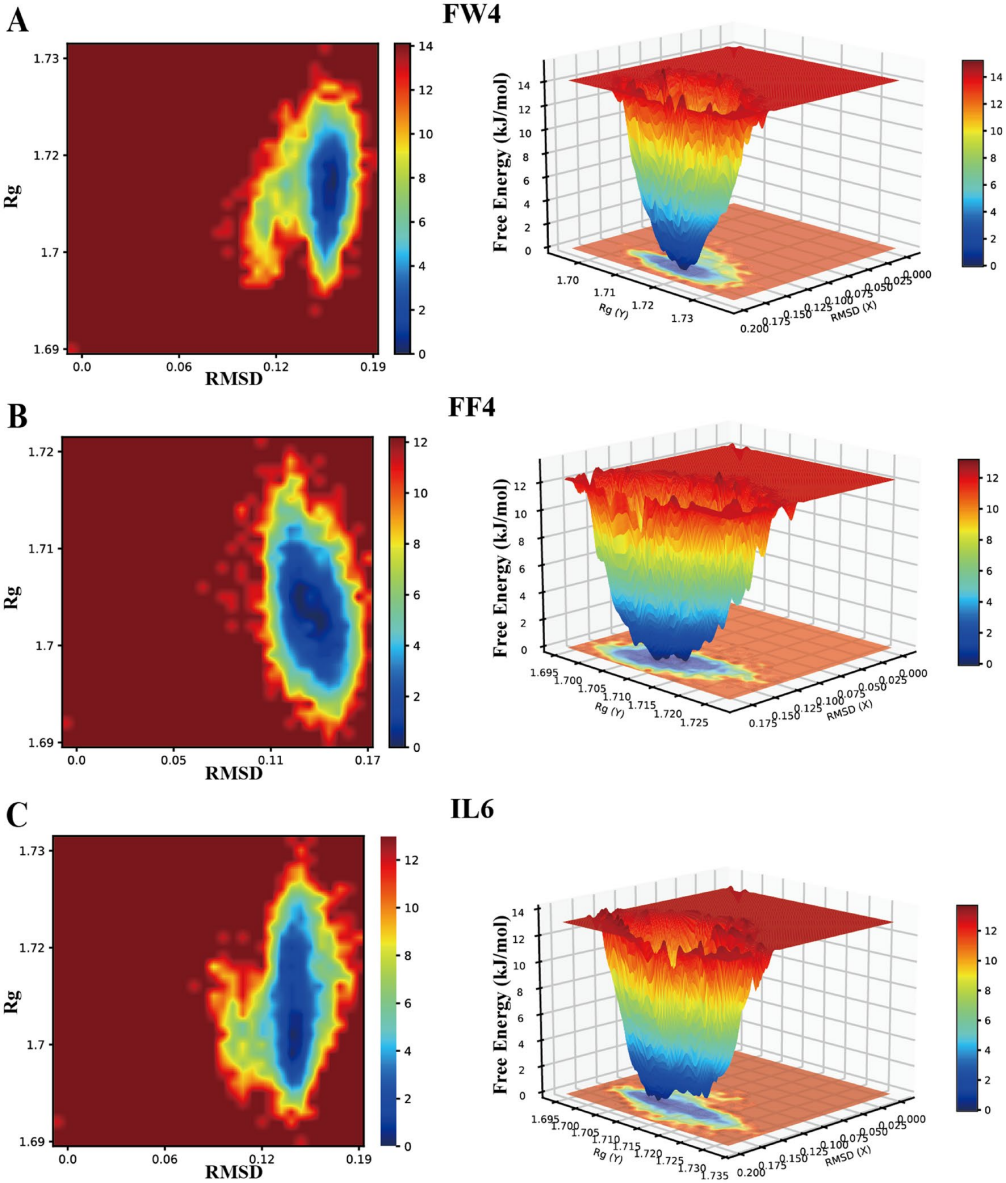
Conformational space energy landscapes of peptide‐Keap1 complexes. (A–C) 2D (left) and 3D (right) free energy landscapes of three peptides bound to Keap1.

**FIGURE 11:**
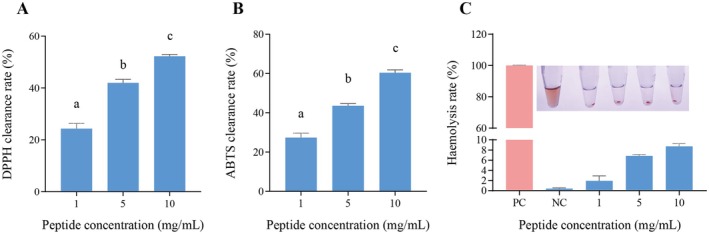
IL6 antioxidant activity and biological safety. (A, B) The scavenging rates of IL6 on DPPH radicals (A) and ABTS radicals. (C) Hemolytic activity of SCP on mouse red blood cells.

To systematically analyze the energy distribution characteristics of complexes in conformational space, two‐dimensional and three‐dimensional free energy landscapes were constructed, and the free energy changes of conformational evolution were quantitatively characterized based on Rg and RMSD. Smaller and more stable RMSD and Rg values indicate smaller conformational fluctuations, more stable binding conformations, and stronger binding affinity to Keap1 (Jiang et al. 2025). The results demonstrated that the free energy conformations of Keap1‐IL6, Keap1‐FF4 and Keap1‐FW4 complexes corresponded to RMSD 0.13–0.14 nm/Rg 1.698–1.704 nm, RMSD 0.12–0.16 nm/Rg 1.702–1.707 nm and RMSD 0.15–0.17 nm/Rg 1.713–1.717 nm, respectively (Figure 10A–C). Notably, the Keap1‐IL6 complex exhibited the narrowest RMSD range and the lowest average Rg value, indicating the most stable conformation and the tightest binding. Furthermore, IL6 outperformed FF4 and FW4 with superior water solubility, as well as stronger resistance to digestive hydrolysis conferred by its proline residue (Sun et al. 2022), endowing it with greater application potential in oral functional foods. In contrast, FF4 and FW4 were limited by high hydrophobicity (Table S2) which hindered activity validation. Therefore, we further synthesized the IL6 peptide and found that it exhibited high DPPH and ABTS radical scavenging activities. Both scavenging rates exceeded 50% at a concentration of 10 mg/mL, while its hemolytic activity remained below 10% (Figure 11A–C). Based on the above results, we confirmed that IL6 is the peptide with the highest antioxidant activity from *S. chinensis*.

## 4 Discussion

Natural antioxidants exhibit safe and application potential in alleviating oxidative stress induced damage, including UVB induced skin oxidative damage, due to their strong free radical scavenging capacity, excellent biocompatibility, and low toxic side effects. As a traditional Chinese plant, *S. chinensis* has been confirmed to possess multiple effects such as immunomodulation, anticancer activity, and hypoglycemic activities (Li et al. 2018), yet research on its skin antioxidant protection and systematic characterization of its peptide sequences with high antioxidant activity remains insufficient. This study demonstrated that SCP exerts favorable in vitro antioxidant effects, with its scavenging activities against DPPH, ABTS, and hydroxyl radicals enhancing with increasing concentration in a dose‐dependent pattern, consistent with observations in other antioxidant peptides (Praseatsook et al. 2025). *Schisandra sphenanthera*, another major medicinal plant of the same genus, showed significantly weaker DPPH and hydroxyl radical scavenging capacities than SCP (Zhao et al. 2024). Meanwhile, SCP showed equivalent DPPH radical scavenging capacity to GSH at 8 mg/mL. While its direct radical scavenging activity was inferior to that of GSH at lower concentrations, SCP could directly neutralize free radicals and regulate the Keap1‐Nrf2 antioxidant pathway. In contrast, GSH exerts antioxidant activity solely via direct free radical reduction, and suffers from low tolerance to gastrointestinal digestion and poor stability (Yin et al. 2025), while SCP has excellent processing and gastrointestinal stability as well as favorable biosafety, which renders its application value in functional food development far higher than that of GSH. In addition, SCP exhibited repair capacity in UVB induced oxidative damage of HaCaT cells. It reversed 50% of cellular damage, which was characterized by increased activities of superoxide dismutase (SOD) and catalase (CAT) and reduced malondialdehyde (MDA) levels and oxygen species (ROS) content at a dose of 100 μg/mL. Consistently, at concentrations exceeding 100 μg/mL, SCP not only rescued UVB‐induced damaged HaCaT cells but also exhibited proliferative activity that elevated cell viability above that of the untreated control group. This proliferative effect of SCP has also been validated in 293 T cells (Li et al. 2022). SCP exerts its protective effect against UVB‐induced HaCaT cell damage mainly derived from its direct neutralization of ROS via hydrophobic amino acids, as well as its stable binding to Keap1 protein to activate the Keap1/Nrf2‐ARE antioxidant signaling pathway (Bao et al. 2025; Kong et al. 2023). The antioxidant activity of SCP was also validated in HepG2 cells subjected to hydrogen peroxide induced oxidative damage (Wang, Zhang, et al. 2025).

Biosafety and stability are core prerequisites for the wide application of bioactive peptides. The former ensures that peptides do not exert adverse effects on normal physiological functions while exerting antioxidant effects, and the latter is the key to maintaining bioactivity under environmental factors such as processing and storage as well as physiological conditions including enzymatic hydrolysis and acid base fluctuations in vivo (Zaky et al. 2021). This study confirmed that SCP retained over 60% DPPH radical scavenging rate under various conditions. Although high salt environments disrupt peptide spatial structure, SCP still maintained more than 70% antioxidant activity at 8 mg/mL NaCl (Gallego et al. 2018). High temperature usually destroys the secondary structure of peptides (Zhao et al. 2020), yet SCP showed stronger antioxidant activity at 100°C than at 60°C. It is proposed that high temperature exposes active amino acid residues such as tryptophan (Trp), alanine (Ala), methionine (Met), and glycine (Gly), enhancing their binding capacity to free radicals and thereby improving antioxidant efficacy (Liu et al. 2020). In addition, compared with neutral conditions, SCP exhibited higher antioxidant activity in acidic environments rather than in alkaline environments, which was verified in in vitro digestion systems simulating gastric and pancreatic juices, indicating that SCP is more suitable for food processing scenarios with high salt, high temperature, and acidity. Regarding biosafety, previous studies have demonstrated that substances with a hemolysis rate exceeding 5% exhibit hemolytic effects and potential toxicity (Wang, Xu, et al. 2025). However, SCP maintained a hemolysis rate below 1% even at a high concentration of 8 mg/mL. Meanwhile, we also confirmed that SCP exerted a positive effect on promoting cell proliferation when treated at concentration of 100 μg/mL or higher.

Structural properties are the primary determinants of the antioxidant activity of peptides. Small molecule peptides are more likely to expose active residues to capture free radicals, and physicochemical properties such as hydrophobicity and isoelectric point regulate the interaction mode between peptides and free radicals (Lin et al. 2024). Previous studies have confirmed that peptides with a molecular weight of < 3 kDa exhibit stronger antioxidant activity (Zou et al. 2016). We also demonstrated that such peptides account for over 99% of SCP, which serves as an important structural basis for its high antioxidant activity. Further screening of SCP identified 63 potential bioactive peptides, most of which had an isoelectric point around five. This characteristic is directly related to the proportion of acidic amino acids, which also explains why SCP maintains high activity in an acidic environment (León‐López et al. 2019). In addition, peptides rich in hydrophobic amino acids such as phenylalanine (Phe) and leucine (Leu) can significantly enhance their own hydrophobicity, which facilitates binding to lipid free radicals and improves scavenging efficiency (Zhu et al. 2014). Notably, only 2 peptides contained trypsin cleavage sites, suggesting that SCP can maintain bioactivity in pancreatic juice. However, most antioxidant peptides exhibit an inherent bitter taste, which severely limits their direct application in the food industry. This bitterness is predominantly derived from the high proportion of hydrophobic and aromatic amino acids in the peptide sequence, and this amino acid composition is also a shared structural feature of highly active antioxidant peptides (Elhadad and Wu 2025). Fortunately, existing technical strategies, including sweetener supplementation, encapsulation, and glycosylation modification, can significantly alleviate the adverse sensory impacts of bitterness on food products (Mirzapour‐Kouhdasht et al. 2023; Yu et al. 2024).

Amino acid composition and spatial conformation represent additional critical determinants of the antioxidant activity of peptides. The binding affinity and conformational stability of peptide‐ligand complexes, primarily mediated by non‐covalent interactions such as hydrogen bonds and van der Waals forces, directly dictate the antioxidant efficacy of peptides (Bissantz et al. 2010). Studies have shown that peptides rich in hydrophobic amino acids (Val, Ala, Tyr, Pro, Met, Trp, Leu), basic amino acids (His, Arg), and aromatic amino acids (Tyr, Phe, Trp), which can stably bind to free radicals via hydrogen bonds, hydrophobic interactions, and other intermolecular forces, exhibit strong antioxidant activity (Tawalbeh et al. 2023). Correspondingly, Phe, Trp, and Leu, the most frequently occurring residues in the interaction between the 63 potential bioactive peptides and DPPH/ABTS radicals, can stably bind to free radicals through hydrogen bonds and hydrophobic interactions, which are the key to SCP exerting its antioxidant effects.

Beyond direct free radical scavenging, antioxidant peptides can also target Keap1 to competitively bind its Nrf2‐binding domain, dissociate the Keap1‐Nrf2 complex, and further activate the antioxidant pathway (Kim et al. 2024). Consistent results from molecular docking and molecular dynamics simulations indicated that FF4, FW4, and IL6 exhibited optimal binding stability to Keap1. Among them, Val‐418 and Val‐514 were reported as key binding sites, which can mediate stable binding between peptides and Keap1 through hydrogen bonds and hydrophobic interactions (Jin et al. 2025). Notably, Keap1 possesses a large binding pocket (Sinha et al. 2025), which facilitates the formation of extensive hydrogen bonds, hydrophobic interactions, and van der Waals forces. In contrast, DPPH and ABTS radicals are extremely small molecules with very limited surface area, which can only form non‐covalent bonds with 1–2 amino acid residues, resulting in relatively low absolute binding affinity (Guo et al. 2024). Furthermore, there was a high overlap in the fluctuation trends of amino acid residues of the Keap1‐FW4 and Keap1‐IL6 complexes, indicating that the patterns of protein structural changes induced by their binding to Keap1 are similar. This finding further suggests that the two peptides may have similar mechanisms of action on Keap1 (Venugopal et al. 2022). Free energy landscape analysis further confirmed that the Keap1–IL6 complex exhibited a smaller and more stable radius of gyration (Rg) and root‐mean‐square deviation (RMSD) in the low‐energy region. Meanwhile, IL6 showed high DPPH and ABTS radical scavenging activities, combined with its favorable physicochemical properties, indicating that IL6 is the key antioxidant peptide from *S. chinensis*.

In conclusion, this study confirms that SCP exhibits excellent antioxidant activity, as evidenced by high DPPH, ABTS, and hydroxyl radical scavenging rates, significant repair effects on UVB induced HaCaT cells oxidative damage, as well as stability and biosafety. A total of 8451 peptides were identified via peptidomics, mainly small molecule peptides (< 1 kDa). Further screening yielded 63 potential bioactive peptides, 8 of which showed strong binding activity to DPPH and ABTS. Among them, FF4, FW4, and IL6 had the optimal binding energy to Keap1, a key molecule in the antioxidant pathway, as revealed by molecular docking analysis. Their binding mainly relies on hydrogen bonds, with Val, Gly, and Arg serving as core interacting residues. MD simulations further verified the stable binding characteristics between these three peptides and Keap1, and clarified the Rg and RMSD ranges corresponding to the minimum free energy and most stable conformation of each complex. Notably, IL6 was confirmed to possess the most potent antioxidant capacity among the three candidates. This study provides a theoretical basis for the development and application of SCP in antioxidant preparations.

## Author Contributions

**Xingyu Xiao:** resources, software, investigation, writing – review and editing. **Shuo Zheng:** investigation, writing – original draft, writing – review and editing. **Tong Su:** visualization, methodology. **Linlin Hao:** investigation, conceptualization, project administration, funding acquisition. **Qinchuan Lv:** investigation, visualization. **Huayi Lu:** investigation, writing – review and editing, funding acquisition. **Jiayuan Fang:** supervision. **Yi Li:** software, investigation.

## Funding

This work was supported by the Jilin Provincial Scientific and Technological Development Program (20220204057YY).

## Ethics Statement

The authors have nothing report.

## Conflicts of Interest

The authors declare no conflicts of interest.

## Supporting information


**Table S1:** Bioactivity profiles of *Schisandra chinensis* candidate peptides.

**Table S2:** Physicochemical properties of *Schisandra chinensis* candidate peptides.

**Table S3:** Molecular docking binding characteristics of *Schisandra chinensis* candidate peptides with DPPH/ABTS.

## Data Availability

The authors confirm that the data supporting the findings of this study are available within the article and Tables S1–, S3.
